# The Differential Impact of COVID-19 Lockdown on Sleep Quality, Insomnia, Depression, Stress, and Anxiety among Late Adolescents and Elderly in Italy

**DOI:** 10.3390/brainsci11101336

**Published:** 2021-10-11

**Authors:** Giulia Amicucci, Federico Salfi, Aurora D’Atri, Lorenzo Viselli, Michele Ferrara

**Affiliations:** 1 Department of Biotechnological and Applied Clinical Sciences, University of L’Aquila, 67100 L’Aquila, Italy; giulia.amicucci@uniroma1.it (G.A.); federico.salfi@graduate.univaq.it (F.S.); aurora.datri@univaq.it (A.D.); lorenzo.viselli@graduate.univaq.it (L.V.); 2 Department of Psychology, “Sapienza” University of Rome, 00185 Rome, Italy

**Keywords:** lockdown, COVID-19 pandemic, late adolescents, elderly, sleep, mental health, stress

## Abstract

The restraining measures due to the COVID-19 outbreak deeply affected the general population’s sleep health and psychological status. The current literature proposes young and older people as two particularly at-risk groups. However, the differential impact of the lockdown period in these specific age categories needs to be disentangled. Through a web-based survey adopting validated questionnaires, we evaluated and compared sleep quality/habits, insomnia, perceived stress, depression, and anxiety symptoms of Italian late adolescents (*n* = 670; mean age ± SD, 19.38 ± 0.74, 18–20 years) and elderly (*n* = 253; 68.18 ± 2.79, 65–75 years). Young respondents reported more severe insomnia symptoms, worse subjective sleep quality, longer sleep latency, higher daytime dysfunction, and a more prevalent disruption of sleep habits (bedtime, get-up time, nap) than the elderly. On the other hand, older participants showed shorter sleep duration, lower habitual sleep efficiency, and greater use of sleep medications. Finally, the younger population displayed higher levels of depression and perceived stress. Our findings indicate that the lockdown period had more pervasive repercussions on sleep and the mental health of late adolescents. The implementation of supportive strategies is encouraged for this vulnerable population group.

## 1. Introduction

The spread of SARS-CoV-2 in China during December 2019 quickly led to the development of a pandemic [1]. Italy was the first European country to impose a total lockdown (9 March–4 May 2020), adopting unprecedented restrictive measures to reduce the infection and death rates. Social isolation, quarantine, mandatory closure of schools, and most work activities deeply compromised the overall mental health and psychological well-being of Italian citizens [2,3]. The pandemic emergency was marked by increased stress and an exacerbation of anxiety and depressive symptoms among the general population. Moreover, the COVID-19 outbreak pervasively altered sleep patterns, as evidenced by poorer sleep quality and increased insomnia levels [3,4,5].

During the lockdown, the elderly could be considered as one of the most at-risk population groups. As far as sleep is concerned, older people normally exhibit several alterations of sleep patterns due to the physiological aging process [6]. Moreover, the elderly’s sleep problems can be exacerbated by an increasing prevalence of multimorbidity, polypharmacy, and psychosocial factors [7]. Several studies showed that older adults report poor sleep quality [8], with insomnia as the most common sleep disorder [9,10]. Notably, the prevalence of sleep disturbances is higher in the elderly than in the young population [8,10].

The pandemic-related factors, including home confinement, social isolation, and the fear of contracting the virus, could directly impact the sleep of older adults [11]. However, the relationship between sleep and COVID-19 outbreak in the elderly is still unclear. Recent studies indicated that older age represented a protective factor for sleep health [3,12]. During home confinement, the younger population reported lower sleep quality [13], increased occurrence of sleep problems [14,15], and worsening of existing sleep problems [14] compared to older people. Conversely, other investigations identified advanced age as a risk factor for sleep disturbances [16], being associated with a decline of sleep quality [17], especially in older individuals with depressive and anxiety symptoms [18]. A recent study also showed that older age represented a significant predictor of a higher association between sleep problems and psychological distress [19]. Moreover, social isolation could exacerbate feelings of loneliness which, in turn, could compromise sleep and psychological health among the older population [20,21].

The fear of contagion could be considered a further factor that can negatively affect general well-being due to the high morbidity and mortality rates in the elderly [22]. However, several studies showed that the older population reported less psychological distress during the COVID-19 outbreak (for a review, [23]), exhibiting higher levels of resilience than the younger counterpart [24]. Although these results may be counterintuitive, it has been shown that late adolescents are more prone to suffer the repercussions of the lockdown on their mental health, representing the less resilient population group than previous generations [25]. Young people exhibited even lower resilience during the pandemic when compared with normative data [26]. Consistently, they seemed to represent an age group strongly affected by the current emergency. Several studies indicated that the younger population showed higher levels of stress, anxiety, and depression [2,27], also ascribable to the deeply disrupted education and social life [28].

In the present study, through a web-based survey, we investigated sleep quality/habits, insomnia, depression, perceived stress, and anxiety symptoms of two particularly at-risk age population groups, that is, late adolescents (18–20 years) and elderly (65–75 years) during the lockdown of Spring 2020 in Italy. We hypothesized to highlight differences in sleep quality/habits, insomnia, and mental health between late adolescents and elderly. However, the above-reported articulated literature does not allow hypothesizing the direction and the extent of the effects of the lockdown on these specific age groups. Therefore, we exploratively compared these two Italian samples to identify specific age-related vulnerabilities for sleep disturbances and psychological problems during the home confinement due to the COVID-19 outbreak. Determining the most vulnerable population categories is essential for designing and implementing specific interventions to mitigate the potential repercussions on sleep and mental health.

## 2. Materials and Methods

### 2.1. Participants

The present study belongs to a larger research project aimed at identifying and understanding the sleep-related and psychological consequences of the COVID-19 pandemic on the Italian population [4].

A total of 13,989 Italian citizens took part in a web-based survey during the lockdown period due to the first contagion wave of COVID-19 (25 March–3 May 2020).

According to the present study’s objective, two subsamples of participants were selected from the whole sample. The first subsample comprised 253 elderly subjects aged 65 to 75 years (mean age ± SD, 68.18 ± 2.79, 104 males), while the other subsample consisted of 670 late adolescents (18–20 years, 19.38 ± 0.74, 182 males).

### 2.2. Procedure

The survey was disseminated through a snowball technique, using social networks (Facebook, WhatsApp, Instagram, and Twitter). Firstly, information about age, gender, the perceived impact of the lockdown on sleep quality, the occurred changes in bedtime, get-up time, and nap habits were collected. Secondly, the survey comprised an evaluation of sleep quality, the severity of insomnia symptoms, and chronotype, using a set of validated questionnaires.

The Pittsburgh Sleep Quality Index (PSQI) [29] was used to assess sleep quality. It is a 19-item questionnaire that includes the evaluation of seven different sleep dimensions: sleep quality, sleep duration, sleep latency, habitual sleep efficiency, sleep disorders, the use of sleep medications, and daytime dysfunction. A higher total score (range 0–21) indicates more severe sleep problems. A cut-off score of 5 is a valid indicator of poor sleep quality [30].

Insomnia symptoms were evaluated using the Insomnia Severity Index (ISI) [31], a 7-item clinical instrument to assess the severity of insomnia condition (range 0–28). A score ranging between 0 and 7 denotes no significant insomnia, between 8 and 14 subthreshold insomnia, from 15 to 21 moderate insomnia, and a total score of 22–28 identify severe insomnia condition [32].

The Morningness–Eveningness Questionnaire-reduced version (MEQr) [33] is a validated 5-item questionnaire (range 4–25) used to identify circadian typologies (4–10: evening-type; 11–18: neither-type; 19–25: morning-type).

Finally, we collected information about depression, perceived stress, and anxiety symptoms, using the Beck Depression Inventory-second edition (BDI-II) [34], the Perceived Stress Scale-10 (PSS-10) [35], and the state-anxiety subscale of the State-Trait Anxiety Inventory (STAI-X1) [36], respectively.

The BDI-II is a 21-item questionnaire widely used in clinical practice to evaluate depressive symptoms (range 0–63). Validated cut-off scores are used to identify the severity of depression conditions (0–13: no or minimal depression, 14–19: mild depression, 20–28: moderate depression, and 29–63: severe depression).

The PSS-10 is a 10-item questionnaire used to evaluate thoughts and feelings referred to stressful events. A higher total score (range 0–40) identifies more significant perceived stress.

The STAI-X1 is a 20-item scale included in the Cognitive Behavioral Assessment battery 2.0 [37]. A higher total score (range 20–80) denotes greater state anxiety.

In order to guarantee greater reliability of the collected responses, the compilation of the last three questionnaires (BDI-II, PSS-10, STAI-X1) was made optional. Of the older respondents, 67.6% and 64.0% completed the BDI-II and the 10-PSS, respectively, while 63.6% completed all the questionnaires. On the other hand, among young respondents, 69.6% and 63.0% compiled BDI-II and PSS-10, respectively, and 61.0% of them also completed the STAI-X1.

### 2.3. Statistical Analysis

The statistical analyses were performed using SPSS v.22 (IBM Corp., Armonk, NY, USA).

The questionnaire scores (PSQI, ISI, MEQr, BDI-II, PSS-10, STAI-X1) of the two subsamples (Elderly, Young) were compared using the Kruskal-Wallis test due to violation of the normality/heteroscedasticity assumptions. The same analysis was applied to bedtime, get-up time, and each sub-component of the PSQI to further understand putative differences in sleep quality/habits between the two groups (Elderly, Young).

We excluded 16 older and 62 young respondents from the analysis on PSQI total score and one of its sub-components (habitual sleep efficiency) due to compilation errors, as respondents reported longer sleep duration than time in bed.

Moreover, we carried out frequency analyses to investigate the proportion within the two groups (Elderly, Young) of the reported impact of the lockdown period on sleep (negative, none, positive) and the changes of bedtime (advanced, unchanged, delayed), get-up time (advanced, unchanged, delayed), and nap habits (increased, unchanged, reduced). Likewise, the same analysis was applied to the PSQI and ISI scores identifying the proportion of poor sleepers and clinical insomniacs through the validated cut-offs. Then, we performed Chi-square tests to evaluate the association between the group membership (Elderly, Young) and the above-mentioned self-report variables.

All the analyses were two-tailed, and the level of significance was set at *p* < 0.05. All *p* values were corrected for multiple comparisons with false discovery rate (FDR) [38]. Eta squared (*ε*^2^) and *Cramer’s V* were computed to provide effect size estimates for Kruskal-Wallis and Chi-square tests, respectively.

## 3. Results

### 3.1. Sleep Variables

**Figure 1:**
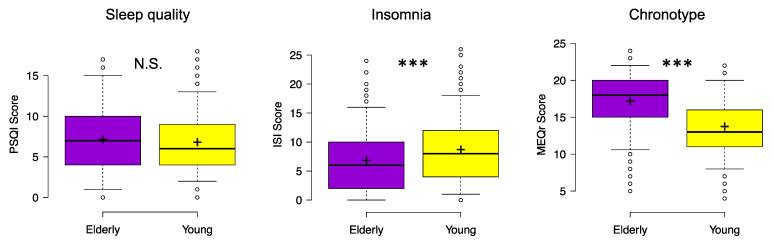
Sleep quality (PSQI), severity of insomnia symptoms (ISI), and inclination to Morningness–Eveningness (MEQr) for elderly and young respondents during the COVID-19 lockdown. Center lines show the medians; box limits indicate the 25th and 75th percentiles; whiskers extend to 5th and 95th percentiles, dots represent outliers; crosses represent sample means. Significant differences of Kruskal–Wallis test between elderly (violet) and young (yellow) participants are indicated with asterisks (*** *p* < 0.001). Abbreviations: PSQI, Pittsburgh Sleep Quality Index; ISI, Insomnia Severity Index; MEQr, Morningness–Eveningness Questionnaire-reduced version; N.S., not significant.

The two groups did not significantly differ in overall sleep quality (Figure 1), as showed by the analysis on PSQI total scores (mean ± SD; Elderly: 7.13 ± 3.95; Young: 6.79 ± 3.33; *χ*^2^ = 0.68, *p* = 0.41, *ε*^2^ < 0.001). However, the comparisons on PSQI sub-components highlighted several significant differences (Table 1). The elderly showed shorter sleep duration, lower habitual sleep efficiency, and greater use of sleep medications than young participants. On the other hand, they reported better subjective sleep quality, shorter sleep latency, and lower daytime dysfunction than late adolescents. Moreover, older respondents reported an earlier bedtime and get-up time.

The analysis of ISI scores highlighted a significant difference between the two groups (*χ*^2^ = 24.43, *p* < 0.001, *ε*^2^ = 0.03). Elderly participants showed lower insomnia symptoms (6.84 ± 5.34) than young respondents (8.69 ± 5.33).

Furthermore, the analysis on MEQr scores displayed a significant difference between the two groups (*χ*^2^ = 150.37, *p* < 0.001, *ε*^2^ = 0.16). Elderly respondents showed a greater inclination to morning chronotype (17.19 ± 3.61) than young subjects (13.74 ± 3.47).

Finally, we observed significant associations between the two groups and the perceived impact of the restraining measures, the reported changes in bedtime, get-up time, and nap habits, and the prevalence of clinical insomnia conditions (Table 2). Prevalence data showed that more than six out of ten late adolescents reported a negative impact of the lockdown period, while a lower rate of older respondents reported a negative impact of the restraining measures.

Moreover, a higher proportion of elderly participants showed unchanged sleep patterns (bedtime, get-up time, and nap habits) than young subjects. Remarkably, three out of four young respondents declared a delayed sleep phase. Finally, older people were characterized by a lower rate of clinical insomnia conditions compared to young people. Chi-square tests did not show a significant association between the two groups and the prevalence of poor and good sleepers.

In the light of the higher proportion of women in the young sample and the well-documented gender differences of sleep problems during the lockdown period [39], we performed control analyses that excluded a possible gender bias in our pattern of results (data not shown).

### 3.2. Psychological Variables

**Figure 2:**
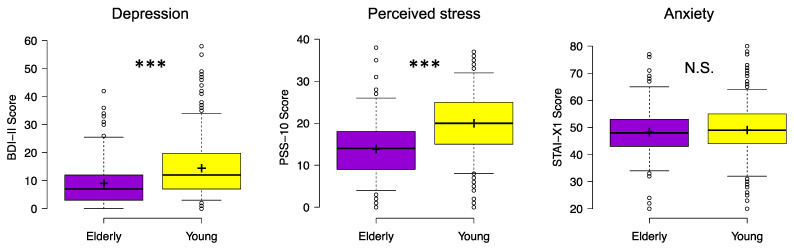
Depression symptoms (BDI-II), perceived stress (PSS-10), and anxiety (STAI-X1) for elderly and young respondents during the COVID-19 lockdown. Center lines show the medians; box limits indicate the 25th and 75th percentiles; whiskers extend to 5th and 95th percentiles, dots represent outliers; crosses represent sample means. Significant differences of Kruskal-Wallis test between elderly (violet) and young (yellow) participants are indicated with asterisks (*** *p* < 0.001). Abbreviations: BDI-II, Beck Depression Inventory-second edition, PSS-10, Perceived Stress Scale-10; STAI-X1, state-anxiety subscale of the State-Trait Anxiety Inventory; N.S., not significant.

There was a significant difference between the two groups in severity of depression symptoms (*χ*^2^ = 54.13, *p* < 0.001, *ε*^2^ = 0.08), and perceived stress (*χ*^2^ = 72.99, *p* < 0.001, *ε*^2^ = 0.12), while anxiety measure did not differ between elderly and young respondents (*χ*^2^ = 1.03, *p* = 0.33, *ε*^2^ = 0.002). As showed in Figure 2, notwithstanding that the two groups did not differ in STAI-X1 scores (mean ± SD; Elderly: 48.20 ± 9.74; Young: 49.0 ± 9.59), older participants showed less severe depression symptoms (BDI-II: 9.01 ± 8.21) and lower stress levels (PSS-10: 13.88 ± 7.10) than late adolescents (BDI-II: 14.45 ± 9.90; PSS-10: 19.95 ± 7.31). Control analyses including gender factor in the models confirmed the differences between the two groups on depression and perceived stress.

## 4. Discussion

According to the study’s hypothesis, we highlighted several differences in sleep and psychological health between late adolescents and older people during the lockdown of Spring 2020 in Italy.

Two-thirds of young participants (64.3%) perceived a negative impact of the restraining measures on their sleep, a greater prevalence than older adults (43.1%). Furthermore, three out of four young respondents showed a delayed sleep phase (bedtime, get-up time). On the other hand, elderly subjects prevalently showed unchanged sleep patterns.

Maintaining the sleep schedule has been suggested as a protective factor to deal with sleep problems during home confinement [40]. In line with this assumption, older people presented lower severity of insomnia than young participants. Conversely, more than half of the late adolescents reported insomnia symptoms from subthreshold to severe extent.

Paradoxically, although the differences in insomnia levels between the two groups, we did not identify significant differences in overall sleep quality. This evidence could be ascribable to the different sleep dimensions covered by the PSQI, whose sum gives rise to the sleep quality measure. Older participants showed shorter sleep duration, lower habitual sleep efficiency, and greater use of sleep medications, in line with the well-documented sleep changes occurring across the lifespan [8,10,41]. We hypothesize that these variables could hardly be affected by the home confinement period in the short term, balancing the outcomes of the other PSQI sub-components. On the other hand, late adolescents showed a worse subjective sleep quality, longer sleep latency, and higher daytime dysfunction, putatively reflecting the more severe insomnia symptoms of this population.

Moreover, a high percentage of late adolescents declared a reduction of naps. Young people’s well-known biological tendency to late sleep timing is typically misaligned with the social clock (academic pressure and social activities) [42,43], configuring the so-called *social jetlag* phenomenon [44]. This situation results in an overall reduction of sleep duration and an accumulated sleep debt during the weekdays among adolescents, leading them to develop compensatory nap habits [45]. As the lockdown period represented an unprecedented condition that unlocked time for sleep for most of the population, we hypothesize that the greater reduction of nap habits of late adolescents reflected the reduction of the social jetlag phenomenon documented among the young population during the period of restraining measures due to COVID-19 outbreak [46].

Although the elderly population exhibits the highest risk of morbidity and mortality during the current pandemic [22], late adolescents seemed to suffer more from the restrictive measures on the psychological side. In line with the current literature on mental health during the pandemic [23], older respondents reported less severe depression symptoms and lower stress levels. However, we did not observe a significant difference in anxiety between the two groups.

Our results are supported by previous research [47], which showed that older people exhibit a higher level of resilience in difficult times than young people, as they experienced greater stressful events during their lifetime, developing better emotional regulation and coping strategies [48,49]. Consistent with this interpretation, a recent study on the Italian population showed that resilience mediated the relationship between pandemic-related stressful events and depression, anxiety, and perceived stress, while age moderated the mediating effect of resilience [24]. Moreover, in line with our findings, another study highlighted that young people presented higher levels of depression, perceived stress, and insomnia than the older counterpart [2].

Furthermore, according to the pre-COVID literature [50], the elderly population reported earlier bedtime and get-up time, and a tendency to morning chronotype. This evidence could constitute a protective factor of older people, as morningness was associated with higher resilience [51,52,53], lower perceived stress [54], and a lower tendency to develop post-traumatic stress disorder (PTSD) [55,56]. In this regard, we recently proposed the evening chronotype as a vulnerability factor during the lockdown period [4,57].

Moreover, the current pandemic emergency impacted younger’s education, contributing to impair mental health of university students [58,59].

Late adolescents were particularly affected by isolation resulting from social distancing [28,60], considering the prominent role of peers and social connections at this stage of life. We hypothesize that these factors could play a role in explaining the greater psychological distress of the young population during the COVID-19 lockdown.

Finally, limited social interactions led to a pervasive increase in the use of digital devices in the hours before falling asleep [61], a deeply rooted habit in our society already before the isolation period among young people. Increased screen exposure has been associated with reduced sleep quality, exacerbation of insomnia symptoms, reduced sleep duration, and longer sleep onset latency during home confinement [61]. Sleep problems could, in turn, negatively affect the psychological well-being of the young population [62]. Of note, excessive screen time was associated with a concomitant higher rate of anxiety and depression symptoms during the lockdown period, especially among young people [63].

To our knowledge, this is the first study on the Italian population aimed at comparing sleep problems and psychological well-being between late adolescents and the older population. However, we must report some limitations. The first one consists of the non-probabilistic sampling technique adopted, as the recruitment of the sample was performed via social networks. This recruitment strategy could limit the generalization of our results to the older population. Moreover, our samples comprised a higher prevalence of women, in particular in the young group. Nevertheless, control analyses confuted a putative gender bias due to the unbalanced gender composition of the two samples. Finally, under-eighteen years old people were not recruited. Future investigations are necessary to clarify the impact of the COVID-19 pandemic on sleep and psychological well-being in this younger population group, considering the strong relationship between sleep and mental health and their influence in the transition toward adulthood [64].

In conclusion, considering the well-known bidirectional relationship between sleep problems and psychological well-being [65], interventions to improve sleep health should be implemented among the young population. Paying attention to sleep hygiene, keeping a stable sleep schedule, and avoiding the overuse of electronic devices before bedtime may prove to be effective strategies to preserve both sleep and psychological health [66]. It is also necessary to implement psychological interventions that, in turn, can support sleep health.

The pandemic continues to plague the daily routine of the general population worldwide. Further research is recommended to evaluate the differential long-term repercussions among late adolescents and the elderly population. This unprecedented period is having a persistent negative impact on the sleep and mental health of the Italian population, as evidenced by the increased perceived stress, and the unchanged prevalence of poor sleepers and moderate/severe depression conditions during the second contagion wave of Winter 2020 compared with the first one [57]. Therefore, we suggest the development of prompt supportive strategies focused on young people, who appeared to be the most vulnerable population group. On the other hand, it is also recommended monitoring older adults’ sleep health and psychological status, as they may develop a concomitant vulnerability over time [24].

## Figures and Tables

**Table 1:** Mean and standard deviation (SD) of the two groups (Elderly, Young), and the corresponding statistical comparisons (Kruskal–Wallis: *χ*^2^, *p*, *ε*^2^), for bedtime, get-up time, and the PSQI sub-components.

	Elderly (65–75 Age)	Young (18–20 Age)	*χ* ^2^	*p*	*ε* ^2^
	Mean ± SD				
Bedtime (hh:mm)	23:46 ± 1:12	1:12 ± 1:41	138.25	<0.001	0.15
Get-up time (hh:mm)	07:55 ± 1:26	9:36 ± 1:41	208.23	<0.001	0.23
PSQI sub-components					
Subjective sleep quality	1.16 ± 0.77	1.39 ± 0.79	15.75	<0.001	0.02
Sleep latency	1.11 ± 1.01	1.57 ± 1.00	36.76	<0.001	0.04
Sleep duration	1.08 ± 0.92	0.50 ± 0.75	93.32	<0.001	0.10
Habitual sleep efficiency	1.11 ± 1.14	0.55 ± 0.89	49.26	<0.001	0.06
Sleep disturbances	1.40 ± 0.65	1.34 ± 0.58	1.37	0.28	0.001
Sleep medications	0.51 ± 1.05	0.23 ± 0.69	14.55	<0.001	0.02
Daytime dysfunction	0.58 ± 0.65	1.15 ± 0.81	93.86	<0.001	0.10

**Table 2:** Prevalence of the lockdown-related perceived impact on sleep, the reported changes of bedtime, get-up time, and nap habits, and the proportion of poor/good sleepers and clinical insomnia conditions within the two groups (Elderly, Young). Chi-square test results are also reported (*χ*^2^, *p*, *Cramer’s V*).

		Elderly (65–75 Age)	Young (18–20 Age)	*χ* ^2^	*p*	*Cramer’s V*
		*n* (%)				
Perceived impact	Negative	109 (43.1)	431 (64.3)	64.42	<0.001	0.26
	None	110 (43.5)	120 (17.9)			
	Positive	34 (13.4)	119 (17.8)			
Bedtime	Advanced	19 (7.5)	36 (5.4)	123.48	<0.001	0.37
	Unchanged	142 (56.1)	134 (20.0)			
	Delayed	92 (36.4)	500 (74.6)			
Get-up time	Advanced	27 (10.7)	57 (8.5)	106.66	<0.001	0.34
	Unchanged	125 (49.4)	116 (17.3)			
	Delayed	101 (39.9)	497 (74.2)			
Nap habit	Increased	33 (13.0)	124 (18.5)	50.19	<0.001	0.23
	Unchanged	196 (77.5)	355 (53.0)			
	Reduced	24 (9.5)	191 (28.5)			
Sleep quality	Poor	151 (63.7)	365 (60.0)	0.97	0.32	0.03
	Good	86 (36.3)	243 (40.0)			
Insomnia	Severe	4 (1.6)	10 (1.5)	13.35	0.004	0.12
	Moderate	22 (8.7)	96 (14.3)			
	Subthreshold	74 (29.2)	245 (36.6)			
	No	153 (60.5)	319 (47.6)			

## Data Availability

The data presented in this study are available on request from the corresponding author.
